# Preoperative prognostic factors associated with postoperative delirium in older people undergoing surgery: protocol for a systematic review and individual patient data meta-analysis

**DOI:** 10.1186/s13643-020-01518-z

**Published:** 2020-11-14

**Authors:** Tayler A. Buchan, Behnam Sadeghirad, Nayeli Schmutz, Nicolai Goettel, Farid Foroutan, Rachel Couban, Lawrence Mbuagbaw, Benjamin T. Dodsworth

**Affiliations:** 1 Ted Rogers Center for Heart Research, University Health Network, 200 Elizabeth Street, Toronto, ON M5G 2C4 Canada; 2 Department of Health Research Methods, Evidence, and Impact, McMaster University, 1280 Main Street West, Hamilton, ON L8S 4K1 Canada; 3 Department of Anesthesia, McMaster University, 1280 Main Street West, Hamilton, ON L8S 4K1 Canada; 4 PIPRA AG, Josefstrasse 219, 8005 Zürich, Switzerland; 5 St. Claraspital, Kleinriehenstrasse 30, 4058 Basel, Switzerland; 6 Department of Anesthesia, Prehospital Emergency Medicine and Pain Therapy, University Hospital Basel, Spitalstrasse 21, CH-4031 Basel, Switzerland; 7 Department of Clinical Research, University of Basel, Schanzenstrasse 55, CH-4031 Basel, Switzerland; 8 Biostatistics Unit/The Research Institute, St. Joseph’s Healthcare, McMaster University, 1280 Main Street West, Hamilton, ON L8S 4K1 Canada

**Keywords:** Postoperative, Prognostic factors, Elderly, Individual patient data meta-analysis

## Abstract

**Background:**

Early identification of patients at risk for postoperative delirium is essential because adequate well-timed interventions could reduce the occurrence of delirium and the related detrimental outcomes.

**Methods:**

We will conduct a systematic review and individual patient data (IPD) meta-analysis of prognostic studies evaluating the predictive value of risk factors associated with an increased risk of postoperative delirium in elderly patients undergoing elective surgery. We will identify eligible studies through systematic search of MEDLINE, EMBASE, and CINAHL from their inception to May 2020. Eligible studies will enroll older adults (≥ 50 years) undergoing elective surgery and assess pre-operative prognostic risk factors for delirium and incidence of delirium measured by a trained individual using a validated delirium assessment tool. Pairs of reviewers will, independently and in duplicate, screen titles and abstracts of identified citations, review the full texts of potentially eligible studies. We will contact chief investigators of eligible studies requesting to share the IPD to a secured repository. We will use one-stage approach for IPD meta-analysis and will assess certainty of evidence using the GRADE approach.

**Discussion:**

Since we are using existing anonymized data, ethical approval is not required for this study. Our results can be used to guide clinical decisions about the most efficient way to prevent postoperative delirium in elderly patients.

**Systematic review registration:**

CRD42020171366.

**Supplementary Information:**

The online version contains supplementary material available at 10.1186/s13643-020-01518-z.

## Background

Elderly patients undergoing surgery are more vulnerable to adverse postoperative outcomes due to advanced age, frailty, and concurrent medical conditions [1]. Postoperative delirium (POD) [2], in particular, is recognized as the most common postoperative complication in the elderly [3], affecting up to 50% of hospitalized surgical patients, POD typically occurs in the early postoperative period and is defined as an acute neuropsychiatric disorder, characterized by fluctuating disturbances in attention, awareness, and cognition. POD is associated with increased morbidity and mortality [4, 5], postoperative cognitive decline and long-term dementia, poor functional recovery, prolonged hospitalization, and increased nursing home admission [6–8].

Besides treating precipitating factors such as infection or pain, there is no simple and effective way to treat POD once it has occurred. Therefore, prevention may be the key to minimizing its occurrence and associated complications. Previous research has shown that delirium may be partially prevented using multicomponent risk intervention strategies [9], which are most effective when targeted at high-risk individuals. A plethora of clinical studies have described individual risk factors for POD [6, 10, 11], and various systematic reviews and meta-analyses have identified several important prognostic factors [10, 12–21]. To date, there has been no IPD meta-analysis to identify prognostic factors for postoperative delirium in older surgical patients and assess their relative prognostic importance.

Using aggregate data for meta-analysis of prognostic factors has important limitations mostly because of the heterogeneous nature of primary studies (e.g., different cut-off levels, diagnostic methods, methods of measurement, and analysis strategies) [22, 23]. Meta-analysis of aggregate data for prognostic studies is limited to study-level estimates of factor-outcome associations that may not be similarly adjusted. In addition, variability in baseline characteristics across studies can only be investigated at between-study level. Therefore, individual patient data (IPD) meta-analysis, in which the raw data from eligible studies are pooled, has been proven to be a superior approach for synthesising prognostic factor studies [24, 25]. There are advantages in using IPD for meta-analyses of prognostic factors including standardizing statistical analyses across included studies, the ability to explore heterogeneity in predictive performance, reducing the risk of over-fitting, and investigating more complex associations and interactions [23, 26].

Early identification of patients at risk for POD is essential as adequately timed interventions may reduce the occurrence of delirium and the related detrimental outcomes [27]. Several pre- and postoperative risk prediction models, using multiple risk factors for POD have been developed in the past [11, 28, 29]; however, these models target highly specific populations and are not widely used in clinical practice. In 2018, the Fifth International Perioperative Neurotoxicity Working Group recommends that all patients above 50 years of age should be informed of the risk of perioperative neurocognitive disorders (including POD) and should receive a baseline cognitive testing before the operation [30]. This systematic review will inform the basis of the first IPD meta-analysis that aims to identify and assess the value of prognostic factors for postoperative delirium in older patients. Subsequently, data will be used to develop a risk prediction model.

## Methods

### Protocol registration and standard reporting

We used the Preferred Reporting Items for Systematic Reviews and Meta-Analyses Protocols checklist when writing this protocol (Additional file 1) [31]. We have submitted and registered our study protocol with PROSPERO (CRD42020171366) and will follow PRISMA-IPD guidance for reporting the results of this review [32].

### Information sources

We will perform systematic searches of MEDLINE, EMBASE, and CINAHL using OVID platform from their inception date to May 2020. A review of the gray literature will also be performed using Google Scholar. We will screen reference lists of included studies and relevant reviews to find additional studies that meet our inclusion criteria. An experienced librarian will refine the searches for individual databases. Additional file 2 provides a draft of our search strategy.

### Study selection

We will include randomized trials, non-randomized studies, case-control studies, retrospective and prospective cohorts, single-arm longitudinal studies that (1) enroll older adults (≥ 50 years) undergoing elective or urgent surgery, (2) assess preoperative risk factors for postoperative delirium, and (3) measure the incidence of delirium (up to 1 week post-surgery) using the Diagnostic and Statistical Manual of Mental Disorders (DSM) criteria by a trained individual, Confusion Assessment Method (CAM) or CAM-ICU, or other validated delirium assessment tools. We will exclude studies that evaluated delirium retrospectively, studies conducted in the ICU setting, and studies that address delirium tremens. In the case of studies investigating heterogeneous patient populations, we will only use data from eligible patients for the analysis. We will not restrict our study selection based on language of publication.

Eligible preoperative risk factors for this review are: age, sex, education, body mass index, type of surgery, frailty, cognitive impairment, comorbidities (such as diabetes, renal insufficiency, cerebrovascular disease, coronary artery disease, myocardial infarction, neurological disorders, dyslipidemia, hypertension, and obstructive lung disease), hypoalbuminemia, psychotropic medication use, history of psychiatric disorders and depression, impairment of activities of daily living, smoking status, alcohol consumption, American Society of Anaesthesiologists physical status classification, Charlson Comorbidity Index, caregiver support, type of anesthesia, and pre-hospitalization.

Pairs of reviewers will independently screen titles, abstracts, and full-text articles of records retrieved through the searches using standardized, pilot-tested forms in Covidence, an online systematic review software (https://www.covidence.org). Any disagreements between reviewers will be resolved through discussion, and if necessary, through participation of a third reviewer.

### Collection of study-level data and data storage

We will contact principal investigators or corresponding authors of eligible studies to inform them about the study and ask if they are willing to share IPD. Those who express an interest in collaboration will be invited to read the protocol and to discuss the requirements for data sharing. The data custodian/representative of the institution will then be invited to sign a data sharing agreement, which specifies the data requested, obligations, ownership of data, terms, and authorship of potential publications. We have developed a specific web link for collaborators with limited access to acquire more information and access the relevant information (https://www.pipra.ch/collaboration.html). We will request authors of eligible studies to provide ethics-approved de-identified data as an encrypted file. All cleaned datasets will be stored in password-protected files on a secured server at McMaster University only accessible to members of the IPD meta-analysis group. All datasets will be in their original formats and will be converted to a master dataset for analysis. Additional file 2 provides a draft copy of our data sharing agreement.

Study authors will be asked to share relevant data regarding candidate predictors and delirium outcomes regardless of whether such data has previously reported in relevant study publication(s). The IPD received will not be used for any other research apart from that described in the data sharing agreement. All collaborators (one representative per study) will be invited to be co-authors on manuscripts describing the principal IPDMA analyses, for which their data contributed, as outlined in this protocol, subject to them meeting the International Committee of Medical Journal Editors (ICMJE) criteria for authorship.

### Risk of bias assessment

We will evaluate validity and bias in studies of prognostic factors using Quality In Prognosis Studies (QUIPS) tool [33]. We will assess the study risk of bias using five domains (study participation, study attrition, prognostic factors, outcome measurement, and study confounding). We will use a modified QUIPS tool to rate each domain as low or high risk of bias as opposed to the original low, medium, and high risk of bias. We will use the individual domains, rated as low, or high risk of bias, to inform the overall risk of bias in each study. Studies with four or five low-risk domains will be considered at overall low risk of bias, and studies with two or more high-risk domains will be considered at overall high risk of bias. Risk of bias assessments will be performed in duplicate and independently.

### Data synthesis and analysis

Data will be inspected for missing data and unusual outliers via range check for all included variables. We will resolve any issues with the data provided through communication and in collaboration with the original authors. Datasets will be accepted in any form, provided that all data are anonymized, and variables and categories are adequately labeled in English. Following satisfactory data control, each dataset will be converted to a common format and variables will be renamed in a consistent manner. The original scales of covariate measurements that are reported will be utilized, where possible. To ensure compatibility across studies, when required, attempts will be made to convert variables to the same scale for all studies. Once each study data is cleaned and standardized, individual study datasets will then be combined to form a new master dataset with a variable added to indicate the original study.

IPD meta-analysis can be conducted using either a one-stage or two-stage approach [34, 35]. Random-effects linear regression fitted with mixed or logistic two-level regression model (with patients nested within studies) will be used in the one-stage approach (using *ipdforst* package in Stata). In this approach, the IPD from all studies will be pooled in a single step, while accounting for clustering of patients within studies. This approach is preferred when few studies or few patients per study are available for synthesis [34, 36].

Two-stage IPD meta-analysis methods can also incorporate covariates and interactions; however, the one-stage approach is likely to be more flexible and practical for modeling of non-linear associations of candidate predictors with outcome. Therefore, our primary analysis will be conducted using the one-stage approach (hierarchical model which will include both study-level and patient-level covariates in the same model). We will use two-stage approach as a sensitivity analysis on the final dataset. All models will allow for random effects and will be estimated using restricted maximum likelihood. Our outcome of interest is postoperative delirium, and we will estimate odds ratios with 95% confidence intervals to quantify the magnitude of outcome-factor associations. There will be no subgroup analyses required. We will use Stata (StataCorp., Release 15.1. College Station, TX) for all data analyses.

### Assessment of certainty in evidence

For assessing our certainty in the prognostic value of the predictors, we will use the Grading of Recommendations, Assessment, Development, and Evaluation (GRADE) approach for prognostic factors [37]. Based on GRADE guidance for prognostic studies because we are addressing the issue of prognosis and not causation, observational studies will start as high certainty but may be rated down based on the limitations in risk of bias, imprecision, inconsistency, indirectness, and publication bias [37, 38]. Certainty can be rated as high, moderate, low, or very low. The GRADE approach is typically applied at the outcome level. Since the focus of our review is prognostic factors for the same outcome, assessment of our certainty will be applied at the level of each individual prognostic factor.

## Discussion

This IPD meta-analysis met the criteria for waiver of ethical approval as defined by McMaster University Ethics Board as it will use existing anonymized data. The prognostic factors identified from our IPD meta-analysis will help to inform the development of a more sophisticated risk prediction model for assessing risk of postoperative delirium in older surgical patients. This will ultimately be used to guide clinical decisions about the most efficient way to prevent postoperative delirium. We will publish the results of our study in an open-access scientific journal and will disseminate our findings in relevant national and international conferences.

## Supplementary Information

**Additional file 1.** PRISMA-P 2015 Checklist.

**Additional file 2.** Search strategy and summary of searches.

## Data Availability

All supplementary data are made available.

## Abbreviations

POD: Postoperative delirium
IPD: Individual participant data
RCT: Randomized controlled trials
NRS: Non-randomized studies of interventions
DSM: Diagnostic and Statistical Manual of Mental Disorders
ICMJE: International Committee of Medical Journal Editors
QUIPS: Quality in Prognostic Studies
GRADE: Grading of Recommendations Assessment, Development and Evaluation
