# Supplementary material for: Preoperative prognostic factors associated with postoperative delirium in older people undergoing surgery: protocol for a systematic review and individual patient data meta-analysis
Source: Syst Rev. 2020 Nov 14;9:261. doi: 10.1186/s13643-020-01518-z (PMC7666505; doi:10.1186/s13643-020-01518-z)
Supplement: Supplementary file 2 — Additional file 2. Search strategy and summary of searches. [file 13643_2020_1518_MOESM2_ESM.docx]

POD Summary of search and strategies

May 5, 2020

| MEDLINE | 7884 |
| --- | --- |
| EMBASE | 9720 |
| CINAHL | 3628 |
| Subtotal | 21232 |
| -dupes | -7035 |
| Total | 14197 |

MEDLINE (Ovid)

Database: OVID Medline Epub Ahead of Print, In-Process & Other Non-Indexed Citations, Ovid MEDLINE(R) Daily and Ovid MEDLINE(R) 1946 to Present

Search Strategy:

--------------------------------------------------------------------------------

1 exp Delirium/ or Confusion/ or exp Cognitive Dysfunction/ or exp Cognition Disorders/ (104782)

2 (delir* or confus* or disorient* or ((cognitive or cognition) adj2 (impairment* or dysfunction* or disorder* or decline* or defect* or deficit*)) or transient mental disorder* or metabolic encephalopath* or acute psycho-organic syndrome* or clouded state* or clouding of consciousness or exogenous psycho* or toxic psycho* or toxic confusion* or acute organic psychosyndrome* or acute brain syndrome*).mp. [mp=title, abstract, original title, name of substance word, subject heading word, floating sub-heading word, keyword heading word, organism supplementary concept word, protocol supplementary concept word, rare disease supplementary concept word, unique identifier, synonyms] (226712)

3 1 or 2 (238913)

4 exp Postoperative Complications/ or exp Perioperative Period/ or exp Postoperative Period/ or Anesthesia Recovery Period/ or exp Surgical Procedures, Operative/ or exp Specialties, Surgical/ or su.fs. (3925104)

5 (surg* or operat* or an?esth* or postoperat* or post operat* or postsurg* or post surg* or perioperat* or peri operat* or perisurg* or peri surg* or resection* or replacement*).mp. [mp=title, abstract, original title, name of substance word, subject heading word, floating sub-heading word, keyword heading word, organism supplementary concept word, protocol supplementary concept word, rare disease supplementary concept word, unique identifier, synonyms] (4290473)

6 4 or 5 (5531187)

7 exp Aged/ or exp Aging/ or Geriatrics/ or Geriatric Nursing/ or Geriatric Assessment/ or Geriatric Psychiatry/ or (old age or older or elder* or geriatric* or aged or aging or frail*).mp. or ((year or years or yr or yrs) adj2 ("65" or "66" or "67" or "68" or "69" or "70" or "71" or "72" or "73" or "74" or "75" or "76" or "77" or "78" or "79" or "80" or "81" or "82" or "83" or "84" or "85" or "86" or "87" or "88" or "89" or "90" or "91" or "92" or "93" or "94" or "95" or "96" or "97" or "98" or "99" or "100" or "101" or "102" or "103" or "104" or "105" or "106" or "107" or "108" or "109" or "110" or "111" or "112" or "113" or "114" or "115" or "116" or "117" or "118" or "119" or "120")).mp. (5845251)

8 3 and 6 and 7 (16510)

9 prognosis/ (501331)

10 exp Epidemiologic Studies/ (2475191)

11 ((cohort or observational) adj3 (study or studies or analy*)).mp. [mp=title, abstract, original title, name of substance word, subject heading word, floating sub-heading word, keyword heading word, organism supplementary concept word, protocol supplementary concept word, rare disease supplementary concept word, unique identifier, synonyms] (566642)

12 (longitudinal or retrospective or cross sectional or prospective).mp. [mp=title, abstract, original title, name of substance word, subject heading word, floating sub-heading word, keyword heading word, organism supplementary concept word, protocol supplementary concept word, rare disease supplementary concept word, unique identifier, synonyms] (2321332)

13 (Follow up adj (study or studies)).tw. (48849)

14 ep.fs. (1647293)

15 risk/ or logistic models/ or protective factors/ or risk factors/ (1016509)

16 (prognosis or prognostic or predict*).mp. [mp=title, abstract, original title, name of substance word, subject heading word, floating sub-heading word, keyword heading word, organism supplementary concept word, protocol supplementary concept word, rare disease supplementary concept word, unique identifier, synonyms] (2264674)

17 or/9-16 (5902309)

18 8 and 17 (8738)

19 exp animals/ not humans.sh. (4695296)

20 exp Animals, Laboratory/ or exp Animal Experimentation/ or exp Models, Animal/ or Rodentia/ or rat.mp. or rats.mp. or mouse.mp. or mice.mp. (3464927)

21 (canine or dog or dogs or monkey or monkeys or rhesus).mp. [mp=title, abstract, original title, name of substance word, subject heading word, floating sub-heading word, keyword heading word, organism supplementary concept word, protocol supplementary concept word, rare disease supplementary concept word, unique identifier, synonyms] (466811)

22 or/19-20 (5749470)

23 18 not 22 (8673)

24 exp Child/ (1892562)

25 (child* or stepchild* or step-child* or kid or kids or girl or girls or boy or boys or teen* or youth* or youngster* or adolescent* or adolescence or preschool* or pre-school* or kindergarten* or school* or juvenile* or minors or p?ediatric* or PICU).ti,ab. (1997070)

26 infant/ or infant, newborn/ or infant, low birth weight/ or infant, small for gestational age/ or infant, very low birth weight/ or infant, extremely low birth weight/ or infant, postmature/ or infant, premature/ or infant, extremely premature/ or birth weight/ (1140336)

27 (perinatal* or antepartum or ante-partum or intrapartum or intra-partum or neonatal* or neo-natal* or postnatal* or post-natal* or pregnan* or fetus* or foetus* or fetal* or foetal* or baby or babies or neonate* or neo-nate* or newborn* or new-born* or infant*).ti,ab. (1334529)

28 or/24-27 (3974045)

29 23 not 28 (7884)

30 limit 29 to english language (7303)

EMBASE (Ovid)

Database: Embase <1974 to 2020 May 04>

Search Strategy:

--------------------------------------------------------------------------------

1 exp Delirium/ or exp Confusion/ or "confusion (uncertainty)"/ or exp cognitive defect/ (530459)

2 (delir* or confus* or disorient* or ((cognitive or cognition) adj2 (impairment* or dysfunction* or disorder* or decline* or defect* or deficit*)) or transient mental disorder* or metabolic encephalopath* or acute psycho-organic syndrome* or clouded state* or clouding of consciousness or exogenous psycho* or toxic psycho* or toxic confusion* or acute organic psychosyndrome* or acute brain syndrome*).mp. (379816)

3 1 or 2 (651938)

4 4 exp Postoperative Complication/ or Perioperative Period/ or exp Postoperative Period/ or exp surgery/ or exp surgical technique/ or su.fs. (5122956)

5 (surg* or operat* or an?esth* or postoperat* or post operat* or postsurg* or post surg* or perioperat* or peri operat* or perisurg* or peri surg* or resection* or replacement*).mp. (5540276)

6 4 or 5 (6881683)

7 exp aged/ or exp Aging/ or exp Geriatrics/ or (old age or older or elder* or geriatric* or aged or aging or frail*).mp. or ((year or years or yr or yrs) adj2 ("65" or "66" or "67" or "68" or "69" or "70" or "71" or "72" or "73" or "74" or "75" or "76" or "77" or "78" or "79" or "80" or "81" or "82" or "83" or "84" or "85" or "86" or "87" or "88" or "89" or "90" or "91" or "92" or "93" or "94" or "95" or "96" or "97" or "98" or "99" or "100" or "101" or "102" or "103" or "104" or "105" or "106" or "107" or "108" or "109" or "110" or "111" or "112" or "113" or "114" or "115" or "116" or "117" or "118" or "119" or "120")).mp. (5383850)

8 3 and 6 and 7 (36319)

9 cohort analysis/ (572734)

10 trend study/ (31778)

11 epidemiology/ or pharmacoepidemiology/ (217462)

12 sensitivity analysis/ (119726)

13 prognosis/ (574038)

14 risk/ (500102)

15 risk factor/ (1022891)

16 ((cohort or observational) adj3 (study or studies or analy*)).tw. (548950)

17 (longitudinal or retrospective or cross sectional or prospective).tw. (2293813)

18 (Follow up adj (study or studies)).tw. (62781)

19 ep.fs. (1013227)

20 (prognosis or prognostic or predict*).mp. [mp=title, abstract, heading word, drug trade name, original title, device manufacturer, drug manufacturer, device trade name, keyword, floating subheading word, candidate term word] (2996910)

21 or/9-20 (6806006)

22 8 and 21 (16130)

23 (rat or rats or mouse or mice or swine or porcine or murine or sheep or lambs or pigs or piglets or rabbit or rabbits or cat or cats or dog or dogs or cattle or bovine or monkey or monkeys or trout or marmoset$1).ti. and animal experiment/ (1057427)

24 Animal experiment/ not (human experiment/ or human/) (2237407)

25 (canine or dog or dogs or monkey or monkeys or rhesus).mp. [mp=title, abstract, heading word, drug trade name, original title, device manufacturer, drug manufacturer, device trade name, keyword, floating subheading word, candidate term word] (457851)

26 or/23-25 (2588829)

27 22 not 26 (16022)

28 exp adolescence/ or exp adolescent/ or exp child/ or exp childhood disease/ or exp infant disease/ (4295719)

29 (adolescen* or babies or baby or boy? or boyfriend or boyhood or girlfriend or girlhood or child* or girl? or infan* or juvenil* or juvenile* or kid? or minors or minors* or neonat* or neo-nat* or neo-nat* or newborn* or new-born* or paediatric* or peadiatric* or pediatric* or perinat* or preschool* or puber* or pubescen* or school or school child* or school* or schoolchild*).mp. [mp=title, abstract, heading word, drug trade name, original title, device manufacturer, drug manufacturer, device trade name, keyword, floating subheading word, candidate term word] (4505669)

30 (pediatric* or paediatric* or infan* or child* or adolescen* or young).jn,jw. (785909)

31 (pediatric* or paediatric* or infan* or child* or adolescen* or young).in. (1808347)

32 (teen* or toddler? or underage? or under-age? or youth*).mp. [mp=title, abstract, heading word, drug trade name, original title, device manufacturer, drug manufacturer, device trade name, keyword, floating subheading word, candidate term word] (150770)

33 or/28-32 (5925023)

34 childhood/ or infancy/ (86818)

35 (perinatal* or antepartum or ante-partum or intrapartum or intra-partum or neonatal* or neo-natal* or postnatal* or post-natal* or pregnan* or fetus* or foetus* or fetal* or foetal* or baby or babies or neonate* or neo-nate* or newborn* or new-born* or infant*).ti,ab. (1586187)

36 or/28-35 (6371754)

37 27 not 36 (13779)

38 limit 37 to english language (13190)

39 limit 38 to conference abstracts (3470)

40 38 not 39 (9720)

CINAHL (EBSCO)

| **#** | **Query** | **Results** |
| --- | --- | --- |
| S24 | S23 Limiters - English Language | 3,628 |
| S23 | S18 NOT S22 | 3,674 |
| S22 | S19 OR S20 OR S21 | 1,562,629 |
| S21 | perinatal* or antepartum or ante-partum or intrapartum or intra-partum or neonatal* or neo-natal* or postnatal* or post-natal* or pregnan* or fetus* or foetus* or fetal* or foetal* or baby or babies or neonate* or neo-nate* or newborn* or new-born* or infant* | 554,313 |
| S20 | child* or stepchild* or step-child* or kid or kids or girl or girls or boy or boys or teen* or youth* or youngster* or adolescent* or adolescence or preschool* or pre-school* or kindergarten* or school* or juvenile* or minors or p?ediatric* or PICU | 1,294,861 |
| S19 | (MH "Child+") | 696,253 |
| S18 | S8 AND S17 | 4,172 |
| S17 | S9 OR S10 OR S11 OR S12 OR S13 OR S14 OR S15 OR S16 | 1,783,563 |
| S16 | prognosis or prognostic or predict* | 521,761 |
| S15 | (MH "Risk Factors") | 175,600 |
| S14 | Follow up N1 (study or studies) | 15,291 |
| S13 | longitudinal or retrospective or cross sectional or prospective | 1,005,404 |
| S12 | ((cohort or observational) N3 (study or studies or analy*)). | 161,379 |
| S11 | (MH "Epidemiology+") | 733,134 |
| S10 | (MH "Epidemiological Research") | 30,935 |
| S9 | (MH "Prognosis") | 80,081 |
| S8 | S3 AND S6 AND S7 | 7,965 |
| S7 | (MH "Aged+") OR (MH "Aged, 80 and Over+") OR (MM "Aged, Hospitalized") OR (MM "Frail Elderly") OR (MH "Geriatrics") OR (MH "Geriatric Psychiatry") OR TX (old age OR older OR elder* OR geriatric* OR aged OR aging OR frail*) OR TX ((year or years or yr or yrs) N2 ("65" or "66" or "67" or "68" or "69" or "70" or "71" or "72" or "73" or "74" or "75" or "76" or "77" or "78" or "79" or "80" or "81" or "82" or "83" or "84" or "85" or "86" or "87" or "88" or "89" or "90" or "91" or "92" or "93" or "94" or "95" or "96" or "97" or "98" or "99" or "100" or "101" or "102" or "103" or "104" or "105" or "106" or "107" or "108" or "109" or "110" or "111" or "112" or "113" or "114" or "115" or "116" or "117" or "118" or "119" or "120")) | 1,633,457 |
| S6 | S4 OR S5 | 1,427,567 |
| S5 | TX (surg* or operat* or anesth* or anaesth* or postoperat* or post operat* or postsurg* or post surg* or perioperat* or peri operat* or perisurg* or peri surg* or resection* or replacement*) | 1,243,652 |
| S4 | ((MH "Postoperative Complications+") or (MH "Postoperative Period") or (MH "Anesthesia Recovery") or (MH "Surgery, Operative+")) | 698,426 |
| S3 | S1 OR S2 | 93,226 |
| S2 | TX (delir* or confus* or disorient* or ((cognitive or cognition) N2 (impairment* or dysfunction* or disorder* or decline* or defect* or deficit*)) or transient mental disorder* or metabolic encephalopath* or acute psycho-organic syndrome* or clouded state* or clouding of consciousness or exogenous psycho* or toxic psycho* or toxic confusion* or acute organic psychosyndrome* or acute brain syndrome*) | 91,504 |
| S1 | ((MH "Delirium") or (MH "Cognition Disorders+") or (MH "Confusion+")) | 41,605 |
